# Lignin Phenol Formaldehyde Resoles Using Base-Catalysed Depolymerized Kraft Lignin

**DOI:** 10.3390/polym10101162

**Published:** 2018-10-17

**Authors:** Pia Solt, Björn Rößiger, Johannes Konnerth, Hendrikus W. G. van Herwijnen

**Affiliations:** 1 Wood K plus-Competence Center of Wood Composites and Wood Chemistry, Kompetenzzentrum Holz GmbH, Altenberger Str. 69, A-4040 Linz, Austria; 2 Department of Material Science and Process Engineering, Institute of Wood Technology and Renewable Materials, BOKU-University of Natural Resources and Life Sciences-Vienna, Konrad Lorenz Str. 24, A-3430 Tulln, Austria; 3 Fraunhofer Center for Chemical-Biotechnological Processes CBP, 06237 Leuna, Germany

**Keywords:** lignin phenol formaldehyde, kraft lignin, base-catalysed depolymerisation, tensile shear strength

## Abstract

Lignin phenol formaldehyde (LPF) resols were produced using depolymerized lignin fractions at various levels of phenol substitution (50 to 70 wt %). To produce monomeric-rich (BCD-oil) and oligomeric (BCD-oligomers) bio-based phenolic compounds, softwood kraft lignin was base-catalysed degraded. These base-catalysed depolymerized (BCD) building blocks were further used to substitute phenol in the synthesis of phenolic resins and were characterized in detail (such as viscosity, free formaldehyde and phenol content, chemical composition, curing and bonding behaviour). The adhesive properties were compared to a phenol formaldehyde (PF) reference resin and a LPF with untreated kraft lignin. The resins synthesized with the two depolymerized lignin types differ significantly from each other with increasing phenol substitution. While with LPF-BCD-oligomers the viscosity increases and the bonding strength is not effected by increasing lignin content in the resin, a reduction of these properties could be observed with LPF-BCD-oil. Furthermore, LPF-BCD-oil showed similar curing behaviour and ultimate strength as the reference LPF. Adhesive bonds made using LPF-BCD-oligomers exhibited similar strength to those made using PF. Compared to the reference resins, it has been demonstrated that modified renewable lignin based phenolic components can be an equally performing alternative to phenol even for high degrees of substitution of 70%.

## 1. Introduction

Synthetic resins such as formaldehyde-based polycondensation adhesive systems are of great importance for the production of wood-based materials. Because of global environmental concerns and a possible scarcity of fossil resources, it is worthwhile to reduce the consumption of synthetic adhesives. During the last decade, a clear increase in research activity to improve the share of adhesives from bio-resources is evident [1]. This transition is also reflected in the prioritization of conferences such as the international conference on wood adhesives (Atlanta, GA, USA, 2017) [2].

Lignin—present in all vascular terrestrial plants—is a complex, amorphous polymer with phenylpropane units linked together by carbon–carbon or ether bonds. It is the second major compound in wood following cellulose and represents 30% of all non-fossil organic carbon on Earth [3,4]. Technical lignins are generated in large quantities as a by-product from chemical wood pulping with around 70 × 10^6^ t/a generated worldwide [3]. However, it is estimated that only 1%–2% of the entire volume is commercially used for products, while the rest is used to generate energy [5]. The dominating technology in pulping is the kraft process with about 89% of total production capacity [3]. Due to the current limitations in lignin application only 75 × 10^3^ t/a of kraft lignin are available on the market [6].

Due to a similar chemical structure, the potential of lignin and in particular technical lignins are attractive biopolymers to replace phenol in the production of PF resins has been an intensively researched topic in material sciences since the 1970s [7,8]. In numerous studies, different lignin types have been investigated in PF resin systems [9,10] and the results have shown that kraft lignin is the most promising alternative. Tejado et al. [10] attributed this advantageous behaviour of kraft lignin towards formaldehyde to its structural and thermal properties but also to its higher number of activated free ring positions and higher molecular weight. Furthermore, for a realistic phenol substitute, its potential high availability should not be neglected.

In contrast to the great number of articles dealing with lignin as phenol substitute published so far, the record of industrial use for wood adhesives is rather poor. Nevertheless, there are some well documented cases of full-scale production experiments based on lignin proportion up to 75% for adhesives (UPM Bio Chemicals [11]) but due to economical, technical and process reasons most of these developments never really reached industrial application [12]. One of the first companies to produce a commercial binder for wood based panel applications using up to 50% lignin is Prefere Resins (Hamina, Finland) [13]. Furthermore, an increasing amount of phenol substitution by lignin to above 50% has been reported to result in a drastically reduction of bonding strengths in previously published works [9,14,15,16].

One of the drawbacks of lignin compared to phenol is its reduced reactivity. Due to the complex chemical structure of lignin, various modification methods—such as methylolation, phenolation or demethoxylation—have already been investigated with the aim of improving the reactivity of lignin towards formaldehyde, with moderate success [3,17]. Furthermore, improvement of lignin homogeneity by solvent fractionation and its influences on the reactivity on LPF resins replacing 50% of phenol was also found to result in lower strength values compared to commercial PF resins [18].

Another promising method to increase the applicability of lignin to generate value added products is the conversion of lignin into smaller fragments through depolymerisation (cracking). The primary purpose of lignin depolymerisation is to convert the complex lignin compound into small molecules and, moreover, increase the chemical reactivity of the degradation products [19,20]. These chemical building blocks provide aromatic and phenolic chemicals from lignocellulosic biomass, which exhibit more similarity to the chemical structure of phenol than lignin does [21]. This property makes depolymerized lignin an extremely interesting alternative to phenol and may allow higher substitution levels through increased reactivity.

The goal of this study is to describe the influence of base-catalysed depolymerized kraft lignin (BCD) on the resin properties of lignin-based phenolic resins. The prepared adhesives, containing up to 70% monomeric-rich (BCD-oil) and oligomeric (BCD-oligomers) bio-based phenolic compounds as phenol substituent, were further characterized in detail and compared to PF reference resin and LPF with untreated kraft lignin in performance.

## 2. Material and Methods

### 2.1. Materials

For the base catalysed depolymerisation, Metsä Fibre Oy (Espoo, Finland) kindly provided precipitated pine kraft lignin, which was generated using carbon dioxide from the black liquor of a pulp mill [22]. The used kraft lignin comprises of 93.1% Klason lignin and 2.9% acid-soluble lignin [22].

Phenol (≥99.5%) and sodium hydroxide (NaOH; 97%) were purchased from Carl Roth GmbH & Co. KG (Karlsruhe, Germany). Industrial formaldehyde (54.5%), which contained 0.55% methanol and <100 ppm formic acid, was provided by Prefere resins Austria GmbH (Krems, Austria) and was stored at 60 °C.

For mechanical testing beech veneer strips with a thickness of 0.58 mm and beech boards with a thickness of 10 mm were acquired at J. u. A. Frischeis GmbH (Stockerau, Austria). Both veneers and boards had an average density of 591 kg/m^3^ and were selected by focusing on homogenous and straight fibre alignment.

### 2.2. Base-Catalysed Depolymerisation

Lignin fractions were generated by base-catalysed depolymerisation (BCD) of purified softwood kraft lignin. For this purpose, the BCD feed solution was prepared by dissolving 7.50 wt % lignin in 3.75 wt % sodium hydroxide solution. Overall 40 kg lignin were processed in a continuous high-pressure pilot plant with a throughput of 1.4 kg dissolved lignin/h, a pressure of 250 bar and a reactor temperature of 320 °C. The average residence time of the alkaline lignin solution was approx. 10 min. Rößiger et al. [23] described a schematic flow diagram of the pilot plant.

The process leads to the hydrolysis of the aryl-aryl ether-bonds and aryl-methyl ether-bonds of the high molecular weight lignin polymers, the formation of low molecular weight phenolic mono-, di- and oligomers and degradation by-products (carbon dioxide and small organic compounds: methanol, formic acid and acetic acid). A detailed description of the proposed mechanisms is outlined in Rößiger et al. [20].

**Figure 1:**
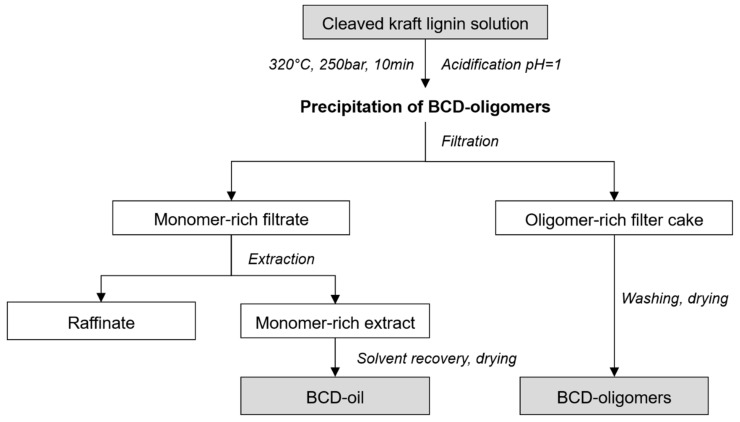
Separation and precipitation process steps of kraft lignin into monomer-rich (base catalysed depolymerised (BCD)-oil) and oligomer-rich (BCD-oligomers) lignin products.

The generated phenolic compounds were then further separated and purified into a monomer-rich base-catalysed lignin oil (BCD-oil) and an oligomer-rich base-catalysed lignin (BCD-oligomers). The summary of separation and purification process flow including the two products is shown in Figure 1. As a final process step, BCD-oligomers were precipitated by acidifying the reaction product solution with sulfuric acid using a semi-continuous down-stream process. A chamber filter press was used for separation and washing of the BCD-oligomers. The filtered reactor water phase was extracted with methyl isobutyl ketone in a counter-current extraction plant with a throughput of 20 L/h. Finally, the isolated base-catalysed lignin fractions were vacuum-dried at 40 °C to constant mass.

### 2.3. Analytical Characterization of Reaction Products

The purified BCD-oligomer filter-cake and the BCD-oil extract were gravimetrically characterized. The yield of the obtained lignin fractions was calculated as the dry mass percentage of the total.

Ash content was determined according to ISO-1762 [24]. 2 g of each lignin sample were dried in a vacuum oven at 40 °C overnight. The sample was subsequently combusted in a ceramic crucible at 530 °C to its mass constant (~6 h) using a muffle furnace AAF 1100 from Carbolite Gero GmbH & Co. KG (Neuhausen, Germany). The remaining residue represents the percentage ash content of the lignin sample.

The specific structural changes in molecular weights were determined by gel permeation chromatography (GPC) using a high-performance liquid chromatography (HPLC) series 1260 from Agilent Technologies (Santa Clara, CA, USA), equipped with GPC columns (AppliChrom ABOA DMSO-Phil-P Pore 250 & 350) and a refractive index detector.

Furthermore, the concentration of individual phenolic, monomeric compounds was quantified by gas chromatography-mass spectrometry (GC-MS) (Agilent GC 7890A and Agilent MSD 5975C). The GC-MS was equipped with a HP-MS5 capillary column (length 30 m, inner diameter 0.25 mm, film thickness 0.33 µm). As internal calibration standard, 1-methylnaphalene was used.

### 2.4. Synthesis of Adhesives

The reference PF resin and untreated LPF reference resin with 50% unfractionated lignin were synthesized according to a recipe by Prefere Resin Oy (Hamina, Finland), using the procedure described by Solt et al. [18].

For the synthesis of LPF with BCD-oligomers, lignin powder was slowly dispersed in 50% NaOH-solution and mixed until a uniform slurry was achieved. Depending on the desired amount of phenol substitution (50%, 60% or 70%), the proportion of lignin in the slurry varies and thus its solid content and slurry viscosity. In the case of BCD-oil, this pre-step was not necessary as the material is already in a liquid state and could be used without further treatment. The LPF resins, both with BCD-oligomers and with BCD-oil, were further produced according to the recipe described in Solt et al. [18]. Because the viscosity of LPF-BCD-oligomers increased very fast during the condensation, the cooking procedure was immediately stopped after adding the final NaOH solution. This resulted in a condensation time of 3.5 h. To ensure a comparison, all LPF resins using BCD-oligomer and BCD-oil lignin were manufactured with the same condensation time.

### 2.5. Physical-Chemical Adhesive Characterization

Solid content of the resins was determined according to EN-827 (2006) standard [25]. Therefore, 2.0 ± 0.2 g of each adhesive was cured in an aluminium container using a ventilated oven at a temperature of 135 °C for 2 h. The solid content was calculated as the dry mass percentage of the total.

The pH value of each adhesive was measured according to ISO-8975 (1989) standard [26]. For this purpose, at least 50 mL of the liquid adhesive was mixed with the same volume of distilled water. The pH value of the mixture was then measured with a glass electrode.

Viscosity of the adhesives was obtained in accordance with ISO-3219 (1993) standard [26] standard at 20 °C. Therefore, 1.3 mL liquid resins were measured with a temperature controlled cone plate rheometer (Bohlin CVO; Malvern Institute Limited, Malvern, UK).

Free formaldehyde content was determined based on ISO 11402 (2005) standard [26]. Therefore, a well-defined amount (9.0 ± 0.2 g) of each resin was dissolved in an isopropanol-water-mixture (3:1). The pH was then adjusted with 0.1 M hydrochloric acid to 3.5, using automatic titration equipment (TitroLine 6000/7000, SI Analytics; Mainz, Germany). Subsequently 25 mL of hydroxylamine hydrochloride solution (10 wt %) was added under continuous stirring. After a reaction of 10 min, the solution was back-titrated to pH 3.5 using 1 M aqueous sodium hydroxide. The calculated free formaldehyde content had to be below a target value of 0.5% ± 0.1%.

Free phenol in the phenolic adhesives were determined on an ICS 5000 HPLC system (Thermo Scientific, Waltham, MA, USA) by Prefere resins Austria GmbH (Krems, Austria). The device was equipped with a UV-detector (271 nm) and Chromolith RP-18e 100 × 4.6 mm column. As external standard phenol was used. The measured value of free phenol had to be below the limit of 0.1 ± 0.03 wt %.

The time required to reach the B-stage of phenolic resin curing (B-time) was measured according to German DIN-8987 (2006) standard [26] using self-constructed equipment. The setup consisted of a heated aluminium plate with a round depression (depth: 5 mm, diameter: 25 mm) in it and a temperature sensor for precise temperature control. 0.5 g of each resin were placed inside the depression at a plate temperature of 140 °C. A glass rod was used to stir the resin for one minute, followed by stirring every minute for 10 s until the B-stage was reached. The B-time was reached when the resin sample was no longer stringy and could be torn off at the end of the glass rod while it was pulled out.

### 2.6. Development of Bonding Strength

To evaluate the development of the bonding strength as a function of hot pressing time a self-constructed hot-press was mounted in a Zwick/Roell Z100 universal testing machine (Zwick GmbH & Co KG, Ulm, Germany). This specific setup was selected in order to allow testing according to ASTM-D7998-15 [27] as originally proposed by Humphrey [28]. The specimens were hot pressed at 120 °C using a relative pressure of 1.5 N/mm^2^. After press times from 30 s to 20 min (0.5, 1, 2, 5, 10, 20 min) the tensile shear strength of the sample was tested with a testing speed of 60 mm/min. Exact method can be found in Solt et al. [18].

### 2.7. Longitudinal Tensile Shear Strength of Solid Wood Joints

Tensile shear strength determination of the adhesives was carried out according to EN-302-1 (2004) [29] and the performance requirements are defined in EN-301 (2017) [30]. In brief, two beech wood lamellas, with a thickness of 5 mm were glued together with a spread rate of 200 g/m^2^. For hot pressing (120 °C at 0.8 N/mm^2^), the pressing time of 20 min was derived from the ascertained curing time results of the automated curing evaluation system. This press time consists of 15 min until almost all systems reached final curing including 5 min through heating time. Samples were cut and treated with two different approaches according to the standard (A1 (dry): 20 °C, 65% rh for 7 days; A2 (wet): additionally stored in water at 20 °C for 4 days and tested in wet condition). The tensile shear strength of all adhesives was determined using the maximum recorded force divided by the overlap area of the joint.

## 3. Results and Discussion

### 3.1. Lignin Characterization

Table 1 displays the characterization of the precipitated kraft lignin and base-catalysed lignin fractions. Gravimetric evaluation of isolated base-catalysed lignin fractions resulted in a recovery of 16 kg BCD-oligomers (40 wt % of lignin) and 6 kg BCD-oil (15 wt % of lignin) out of 40 kg precipitated kraft lignin after pilot scale depolymerisation, separation and purification. The non-isolated 45 wt % of lignin were converted into gaseous and liquid by-products or were lost during the down-stream processing. Current mass balancing results of comparable production campaigns show an allocation of the lignin yield after base-catalysed depolymerisation of 60–70 wt % of BCD-oligomers, 10–15 wt % of BCD-oil, 5–10 wt % gaseous by-products (BCD-gas) and 5–10 wt % small organic compounds. The overall lignin recovery rate is estimated to be increased to 85–95 wt % [23]. In addition to the reduction in molecular weight, ash analyses showed a very low mineral content within the BCD-oligomers of 0.18 wt % and for BCD-oil 0.02 wt %. Besides increased reactivity, this could lead to good applicability of this fraction. GPC showed a strong reduction of the number average/weight average molecular weight of the kraft lignin (*M*_n_/*M*_w_ = 1350/9850 g/mol). The isolated BCD-oligomers possessed a molar mass of 2250 g/mol and furthermore a polydispersity index (PDI) of 3.0. BCD-oil consists of low molecular weights compounds and had the narrowest molar mass distribution (PDI of 2.0) with *M*_w_ of 300 g/mol. Taking coniferyl alcohol with a molecular weight of 180 g/mol as a representative example for a lignin monomer, BCD-oligomers can be classified as oligomer and BCD-oil on average as dimer.

Quantitative GC-MS analysis of phenolic monomers in BCD-oil yields a content of 7.0 wt % guaiacol (*M*_w_: 124 g/mol) and 27.6 wt % catechol (*M*_w_: 110 g/mol). The residual 65.4 wt % of BCD-oil contains unknown or non-volatile mono-, di- and oligomeric phenolic compounds. The general objective of BCD is the reduction of complexities of the natural lignin molecule and increasing its chemical reactivity. These changes in structure can be qualified by analysing the molecular mass distribution. BCD oligomers were obtained as a brown powder. BCD oil is a dark brown liquid with a viscosity of 750 mPa·s.

### 3.2. Physical-Chemical Adhesive Characterization

The results shown in Table 2, namely solid content, pH, viscosity, free formaldehyde, freephenol and B-time, indicate the individual properties of the different lignin based phenolic adhesives using 50–70 wt % phenol substitution by BCD-oligomers and BCD-oil, respectively.

The viscosity measurement showed a strong influence on the amount of lignin in the resin system. While LPF-BCD-oligomers assigned an extreme viscosity increase with increasing lignin content, LPF-BCD-oil shows an opposite behaviour. This characteristic can be attributed to the basic structure of the two lignin types, since BCD-oil comprises low molecular compounds already in the liquid state, whereas BCD-oligomers consists of higher molecular compounds and has to be dispersed in NaOH-solution before incorporating it in the resins synthesis.

Free-formaldehyde content (HCHO) of all LPF resins as well as of the reference resin were in the range between 0.3% and 0.9%. Compared to the standard PF-resole, all LPF-BCD-oligomers resins reached slightly higher values. For LPF-BCD-oligomers 70%, it was not possible to dissolve and further measure the HCHO amount. Furthermore, it can be seen that LPF with BCD-oil shows a significant increase in free formaldehyde with increasing phenol substitution and thus did not reach the selected target value of (≤0.5 wt %) above a substitution degree of 50%. Apparently, the components of BCD-oil have less reactive sites than phenol, resulting in remnant formaldehyde. The amount of free formaldehyde could be reduced by increasing condensation time, which was not done for reasons of comparability. Free phenol contents show again that beside of LPF-BCD-oil, the other resins reached the selected target values below 0.1 wt %.

The B-Time was evaluated at 140 °C. Up to 60% phenol substitution, the BCD oligomers provide resins that are equally reactive as unmodified PF and are more reactive than LPF resins comprising uncracked lignin at a 50% substitution level. At 70% phenol substitution, the resin shows a slower gellation than PF but still similar to the reference LPF. The B-time of LPF-BCD-oil modified resins was in all cases shorter than the B-time of the reference PF resins, albeit at higher levels of free formaldehyde. Since the curing of phenolic resins correlates to the amount of free formaldehyde [31], this could be used as an explanation.

### 3.3. Development of the Bonding Strength

**Figure 2:**
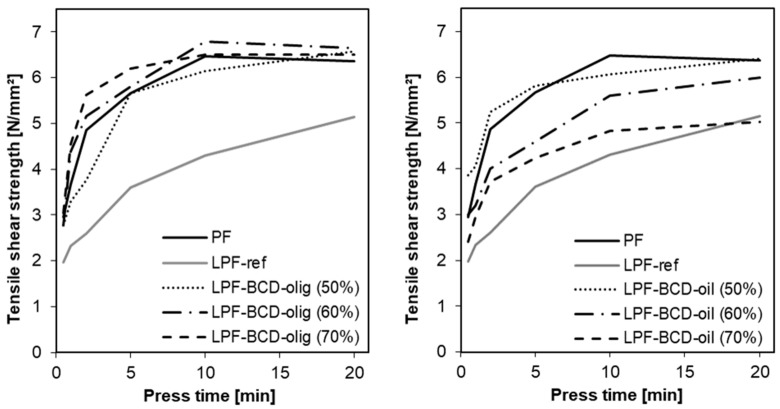
Development of the bonding strength of LPF-BCD oligomers (**left**) and LPF-BCD-oil (**right**).

Measuring the development of the tensile shear strength during hot pressing is a method to investigate the curing properties of binders in the presence of adherents. The development of bonding strength is strongly influenced by a number of key factors such as pressing temperature, pressure and time but also by chemical properties of the adhesive and the adherend materials [32]. Figure 2 shows the bonding strength of all resins as a function of pressing time. Comparing the two reference resins (PF and LPF-ref.), the bonding strength develops much slower when unmodified lignin is used in the adhesive system as partial phenol substitute and does not reach the final strength of the reference phenolic resin.

Using BCD-oligomers (Figure 2), a similar bond strength development as for PF could be achieved. In this case, the ultimate strength of PF resin (6.8 N/mm^2^ with 100% wood failure) was reached with the LPF-BCD-oligomers resins at all three different phenol replacement levels. Furthermore, between the substitution levels no explicit difference can be found in the speed of strength development and the maximum measured tensile shear strength. Even at 70% substitution, a similar performance could be obtained, even though this resin showed a longer B-time. Thus, the BCD-oligomers seem to be reactive oligomers that can be perfectly bonded into the polymer network. For the LPF-BCD-oils a decelerated bonding strength development compared to PF reference was found. The bond strength developed slower as the lignin amount was increased. Especially in case of LPF-BCD-oil 70%, the curve shows a level-off in strength after 10 min pressing time at a value of 4.9 N/mm^2^, representing therefore a 25% lower result compared to the PF reference. Likewise, Ghorbani et al. [9] and other authors found that curing characteristics, assessed by a similar tensile strength development method, showed a reduction in strength development as phenol substitution increased. As already estimated from the higher free formaldehyde content of the resins, the low molecular lignin fragments might possess less free reactive sites, resulting in a less dense and therefore weaker network. Additionally, the higher viscous LPF-BCD-oligomers is expected to remain in the bond line, whereas the lower LPF-BCD-oils might penetrate deeper into the wood structure possibly leading to a partially starved bond line (not verified). Nevertheless, resins containing base catalysed depolymerized lignin outperform the resin containing unmodified lignin, even at higher substitution degree. From this measurement result it can now be deduced that although the LPF-BCD-oil showed an accelerated condensation polymerization reaction in the B-time test, this does not necessarily indicate strength development.

### 3.4. Longitudinal Tensile Shear Strength

**Figure 3:**
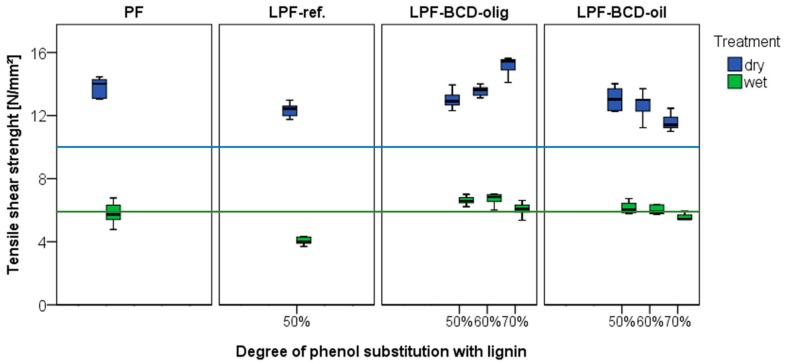
Tensile shear strength of lap joint specimens bonded with all different BCD-lignin based PF resins compared with two reference resins (sample number: *n* = 10). The blue and green lines indicate the EN-301 (2017) [30] standard requirements of A1 (dry) and A2 (wet) treatments for phenolic resins in load bearing timber structures.

Figure 3 displays the lap shear strength of each tested adhesive illustrated in groups. After equilibrating the samples according to treatment A1 (20 °C and 65% rh for 7 days) both PF and all LPF resins (50 to 70 wt % phenol replacement) surpassed the EN-301 (2017) [30] standard requirements of 10 N/mm^2^ for thin bond lines. Interestingly, PF as well as the reference LPF resins bonded samples did not pass the standard tensile shear strength for A2 (additionally stored in water at 20 °C for 4 days and tested in wet condition) treated phenolic resins (6 N/mm^2^), whereas the LPFs with BCD-oligomers and BCD-oil (expect LPF-BCD-oil 70%) did. The lap joint bonded with PF reference resin has an average strength of 14 N/mm^2^ with a high wood failure (median: 90%) and after water storage a strength reduction of 60% with no wood failure. It should be noted that phenolic resins are typically applied where moisture and water-resistant bonding are needed and therefore commercial produced PF resins usually perform well under wet conditions. The weaker performance after treatment A2 could be attributed to the laboratory-scale production but also to the relatively low binder amount (200 g/m^2^). At a solid content of 43.6%, the adhesive joint achieves a solid resin content of 87 g/m^2^, which additionally complicates a homogeneous adhesive distribution. For the case of kraft lignin used in the form of a precipitated lignin without any further treatment in phenolic resins, the tensile shear strength—both wet and dry—is clearly below the PF resoles. As expected from the results of curing strength development, also final strength of LPF-BCD-oligomers show similar tensile shear strength as PF-reference. An apparent trend of increasing strength with increasing lignin content for LPF-BCD-oligomers could be observed, which, however can be neglected due to almost 100% wood failure for all degrees of substitution. As this strength value indicates the strength of the wood substrate, the value is not appropriate for differentiation between adhesive performance [33]. LPF-BCD-oils in turn exhibit a reduction in strength with a higher degree of substitution, combined with a lower proportion of wood failure (LPF with 50% BCD-oil: 90% wood failure; with 70% BCD-oil: 50% wood failure). A reduction in cohesive strength of the bond line can be assumed, while still meeting standard requirements for dry conditions. A comparison of the wet stored samples displays that both lignin types (BCD-olig., BCD-oil) show a comparable strength value for all lignin amounts (between 5.5 and 6.7 N/mm^2^) but higher wet and dry strength level than LPF-reference. This observation for depolymerized kraft lignin is in line with the conclusion of Ma et al. [34]. They indicated that depolymerized lignin—in this case oxidative—improves the bonding strength of LPF resins compared to using the same amount of unmodified kraft lignin (50%). Furthermore, an increasing lignin content for LPF resins to more than 50% has been repeatedly reported to result in a drastically reduction in bonding strengths [9,14,15,16,35]. The result present here, suggested that base catalysed depolymerized lignin represents an appropriate phenol substituent, allowing high amount of phenol substitution.

## 4. Conclusions

Kraft lignin was cracked into two fractions using a base-catalysed depolymerisation process to gain monomer-rich and oligomeric lignin units. These cracked lignins were further used to produce lignin-based phenolic resins with 50–70 wt % substitution of phenol by lignin and compared with untreated kraft lignin based phenolic resins and phenol formaldehyde reference resins. The results gained demonstrated that base-catalysed depolymerisation lignin oligomers (BCD-oligomers) can successfully substitute phenol in corresponding resins at high substitution levels of up to 70% without biasing final dry or wet tensile shear strength. BCD-oligomers showed consistently better performance compared to BCD-oil. Unique to this approach is that even for the high degree of phenol substitution the speed of bond strength development was fully comparable to the PF reference at 120 °C hot pressing temperature. Only the high viscosity of the LPF-BCD oligomer resin poses a challenge for industrial applications, which was not in focus of the present research. By further optimization, cracked lignins have the potential of achieving high performing resin meeting all industrial needs.

## Figures and Tables

**Table 1:** Characterization of the precipitated kraft lignin and base-catalysed lignin fractions.

Lignin Type	Yield	Ash Content ^a^	*M*_n_	*M*_w_	PD	Texture
	(%)	(%)	(g/mol)	(g/mol)		[Viscosity (mPa·s)]
Kraft lignin		1.3	1350	9850	7.3	Powder
BCD-oligomers	40	0.18	750	2250	3.0	Powder
BCD-oil	15	0.02	150	300	2.0	Liquid [750 mPa·s]

^a^ Each value represents an average of 3 samples.

**Table 2:** Resin properties of phenol formaldehyde (PF) and lignin phenol formaldehyde (LPF) resins using base-catalysed depolymerized lignin as phenol substituent.

Resins	Phenol	Solid	pH ^b^	Viscosity ^a^	Free HCHO ^b^	Free Phenol ^b^	B-Time
	Substitution	Content ^a^					140 °C ^a^
	(%)	(%)		(mPa·s)	(%)	(%)	(s)
PF		43.6	12.1	580	0.2	<0.01	92
LPF-ref.	50	46.7	12.4	520	0.4	0.1	120
LPF-BCD-olig.	50	45.7	12.7	540	0.3	<0.1	89
	60	45.0	12.6	1720	0.3	<0.1	96
	70	45.3	12.4	5580	n.a.	<0.1	125
LPF-BCD-oil	50	44.2	12.5	830	0.5	0.3	81
	60	43.7	12.4	450	0.7	0.3	75
	70	43.0	12.4	220	0.9	0.3	71

^a^ Each value represents an average of 3 samples; ^b^ Each value represents an average of 2 samples.
